# Relative importance of temporal and location features in predicting smoking events

**DOI:** 10.1038/s41746-025-01799-5

**Published:** 2025-07-05

**Authors:** Han Yang, Hang Yu, Michael Kotlyar, Sheena R. Dufresne, Serguei V. S. Pakhomov

**Affiliations:** 1 Institute for Health Informatics, University of Minnesota, Minneapolis, MN USA; 2 University of Minnesota, Minneapolis, MN USA; 3 Department of Experimental and Clinical Pharmacology, University of Minnesota, Minneapolis, MN USA; 4 Department of Pharmaceutical Care & Health Systems, University of Minnesota, Minneapolis, MN USA

**Keywords:** Health care, Health care economics, Geography

## Abstract

Pharmacological aids for smoking cessation, such as nicotine gum and lozenges, are most effective when used just before smoking triggers occur. Mobile technology can help by predicting these events and delivering timely reminders. This study examined the predictive value of temporal and spatial features available from smartphones. Thirty-eight participants self-reported 1784 smoking events during up to two weeks of ad-libitum smoking. Temporal features were extracted from timestamps, and spatial features were derived from GPS coordinates using methods such as DBSCAN, K-means, and distance-from-initial location. We trained logistic regression, random forest, and multilayer perceptron models with various half-time intervals (5–30 min). Across all modeling approaches and settings, excluding temporal features led to a substantial decrease in performance, while removing spatial features had a minimal effect. These results suggest that time-related cues are more robust and generalizable predictors of smoking behavior than location, supporting their use in just-in-time smoking cessation interventions.

## Introduction

Smoking remains one of the leading causes of preventable disease and death worldwide, despite decades of public health efforts aimed at reducing its prevalence^1,2^. In the United States, cigarette smoking has been steadily declining; however, as of 2021 about 11.5% of U.S. adults still smoke combustible cigarettes, translating to approximately 28.3 million people^3,4^. The health consequences of smoking are severe, with approximately 480,000 deaths in the United States annually attributable to smoking due to increased risk of a wide range of diseases including lung cancer, heart disease, stroke, and chronic obstructive pulmonary disease (COPD)^5^. Quitting smoking significantly reduces the morbidity and mortality associated with smoking^6–8^. However, cessation remains challenging due to the addictive nature of nicotine, and although a majority of people who smoke express a desire to quit, only a small percentage succeed each year. In 2022, only 8.8% of adults who attempted to quit were successful, underscoring the need for more effective cessation strategies and interventions^9^. Pharmacological aids, such as nicotine gum and lozenges, are commonly used on an as-needed basis to alleviate cravings in individuals trying to quit smoking (either alone or in combination with the nicotine patch)^10–12^. However, the effectiveness of as-needed interventions is likely dependent on timing, since administering them just before smoking trigger exposure (as opposed to after smoking trigger exposure) can maximize their impact on craving severity^10,11,13^.

During a quit attempt, the craving to smoke can be triggered by a variety of cues or situations that prompt an individual to smoke^14,15^. These triggers can include environmental factors, emotional states, social interactions, and specific times of day^14,16–19^. Recognizing and understanding these triggers, particularly in naturalistic settings in which smokers go about their daily lives, is critical for tailoring interventions that can effectively disrupt smoking patterns. A promising approach is the use of just-in-time adaptive interventions (JITAIs), which aim to provide support at the moment when an individual is most vulnerable to smoking^20–22^. JITAIs rely on accurate prediction of smoking events, using real-time data on potential triggers such as location, time, and physiological states, to deliver timely interventions that can prevent a relapse. Mobile technology is well-suited to deliver JIT support as it could potentially be used to both predict moments of high craving and to then provide just-in-time behavioral support or deliver reminders to use pharmacological aids that can be applied on an as needed basis^23–27^.

Previous research has leveraged mobile devices to make significant progress in detecting and predicting smoking events by strategically using various physiological, behavioral, and contextual data sources. Data features include measures of heart rate, skin temperature, physical activity^28–31^, location (determined through Geographical Positioning System (GPS) data) and time-of-day information^19,32–34^. Geofencing specifically, a technique that sets virtual boundaries around specific areas to trigger interventions, has emerged as a potential focus within smoking trigger studies, particularly in identifying and responding to smoking locations in real-time^35^.

Despite significant advances in the detection and prediction of smoking events using physiological and behavioral data, there are notable limitations in previous research. One such gap is the insufficient focus on the specific contributions of location and temporal information to smoking behavior. Many location-based behavioral studies that have incorporated GPS data have done so in a limited manner, often using raw geographic coordinates without applying spatial analysis techniques such as clustering or spatial autocorrelation modeling^26,36–38^. For instance, the direct use of latitude and longitude values, without accounting for dynamic movement patterns or context-specific locations, reduces the ability to capture the complexity of human behavior including smoking triggers^39^. On the other hand, the temporal dimension—specifically the impact of time-related features such as time of day or day of the week—has often been underexplored in smoking behavior research. In contrast to other approaches that have been proposed to predict smoking events (e.g., physiological measures collected by wearable devices, behavioral measures collected using ecological momentary assessment^22,23,29,40^), time and location can be easily and passively measured using smartphones, which most people already own, since temporal and spatial data can be unobtrusively and continuously captured with high-precision. This makes these measures highly suitable for capturing surrogate markers of environmental and situational triggers, offering valuable insights for real-time intervention strategies.

There is limited data regarding the extent to which location information improves the ability to predict smoking events relative to the use of only time information. Although both can be obtained from smartphones, collecting detailed location data raises privacy concerns to a greater extent than just collecting time. Assessing the relative importance of location vs. time data in predicting smoking events could improve our understanding of how environmental and situational factors influence smoking behavior and inform the development of JITAIs.

## Results

Table 1 shows a summary of the basic demographics of all the participants and Table 2 shows the data characteristics. There were no statistically significant differences in age, duration of data collection, mean counts of GPS positions, smoking events, GPS data samples labeled as smoking, and Density-Based Spatial Clustering of Applications with Noise (DBSCAN) geolocation clusters during the pre-quit period between the during–Covid-19 emergency group and the post-Covid-19 emergency group. Detailed statistical testing information is provided in Supplementary Table 1.

**Table 1:** Participant characteristics

Individual-level variables	All Participants (*n* = 38)		During-Covid-19 Emergency Group (*n* = 29)		Post-Covid-19 Emergency Group (*n* = 9)	
	Count (%)	Mean (SD)	Count (%)	Mean (SD)	Count (%)	Mean (SD)
*Age*		41.34 (8.44)		42.03 (8.33)		39.11 (7.89)
*Gender*						
Male	16 (42.11)		15 (39.47)		1 (0.03)	
Female	22 (57.89)		14 (36.84)		8 (21.05)	
*Race/Ethnicity*						
White / Caucasian	25 (65.79)		21 (55.26)		4 (10.53)	
Black / African American	9 (23.68)		6 (15.79)		3 (7.89)	
Hispanic	3 (7.89)		1 (2.63)		2 (5.26)	
Other	1 (2.63)		1 (2.63)		0 (0.00)	
*Timezone*						
Pacific	1 (2.63)		1 (2.63)		0 (0.00)	
Mountain	3 (7.68)		0 (0.00)		3 (7.89)	
Central	15 (39.47)		13 (34.21)		2 (5.26)	
Eastern	19 (50.00)		15 (39.47)		4 (10.53)

**Table 2:** Data characteristics (DBSCAN - Density-Based Spatial Clustering of Applications with Noise)

Individual-level variables	All Participants	During-Covid-19 Emergency Group	Post-Covid-19 Emergency Group
	Mean (SD)	Mean (SD)	Mean (SD)
Duration of data collection (days)	13.65 (3.43)	13.61 (3.81)	13.78 (1.99)
**Timeframe: Entire study period (up to 3 weeks)**			
GPS positions^a^	1019.00 (1545.35)	1268.59 (1739.85)	443.88 (339.91)
Smoking events^a^	90.55 (73.05)	87.55 (71.31)	100.89 (77.86)
All GPS data samples^b,d^	1024.15 (1540.03)	1275.93 (1743.48)	446.67 (350.03)
GPS data samples labeled as smoking^b^	441.95 (613.02)	467.86 (585.79)	219.22 (138.09)
DBSCAN geolocation clusters	3.22 (3.20)	3.31 (3.17)	2.89 (3.28)
**Timeframe: Pre-quit study period (up to 2 weeks)**^c^			
GPS positions^a^	487.37 (900.90)	561.55 (1010.12)	248.33 (253.40)
Smoking events^a^	46.95 (34.50)	44.66 (35.49)	54.33 (29.92)
All GPS data samples^b,d^	492.39 (892.07)	563.07 (1001.33)	264.67 (247.53)
GPS data samples labeled as smoking^b^	197.34 (190.24)	216.86 (209.38)	134.44 (79.77)
DBSCAN geolocation clusters	2.81 (2.16)	2.64 (2.15)	3.44 (2.19)

^a^Counts of raw GPS positions and self-reported smoking events obtained from the participants’ smartphones.

^b^Counts of data samples calculated from the raw GPS positions and self-reported smoking events treated as intervals (based on 15-minute half-intervals) used in predictive modeling.

^c^The predictive modeling analysis was based only on the data collected in the up to 2-week pre-quit study period.

^d^The numbers of all GPS data samples used in predictive modeling is slightly larger than the number of actual GPS samples obtained from the participants’ smartphones because in some rare cases the reported smoking event interval did not align with any recorded GPS positions. In these cases the closest available GPS position immediately outside of the smoking event interval was used.

### Prediction results

Table 3 shows Macro-F1 scores for prediction results across various combinations of location representation methods (DBSCAN, K-means clustering (K-means), and Distance from initial geo-location (DFI)), modeling algorithms (Logistic Regression (LR), Random Forest (RF), and Multilayer Perceptron (MLP)), and different feature sets (all features, location-excluded, time-excluded), and half-time intervals (5-, 10-, 15-, 20- or 30-min) during the pre-quit period of all participants. Across all configurations, models that excluded temporal features consistently experienced a significant drop in performance, highlighting the critical role of temporal information. Notably, RF yielded the highest predictive performance overall, with macro-F1 scores peaking at 87.98% (20-min half-time, DBSCAN). In contrast, the exclusion of location features resulted in only marginal and not statistically significant reductions in performance across all models and time intervals, supporting the conclusion that location contributes less predictive value than time. The detailed test results including t-statistic value, *p*-value, and confidence intervals are provided in Supplementary Table 2.

**Table 3:** Prediction Modeling Results (Macro-F1 Score (unit: %))

Methods combinations between location representation & modeling	All features Mean (SD)	Location Excluded Mean (SD)	Time Excluded Mean (SD)
Half-time interval			
*DBSCAN* + *LR*			
30-min	79.15 (0.11)	78.21 (0.11)	63.20 (0.13)**
20-min	78.84 (0.13)	78.59 (0.13)	64.26 (0.13)**
15-min	75.90 (0.13)	75.15 (0.13)	64.20 (0.13)**
10-min	75.24 (0.12)	75.11 (0.12)	64.05 (0.12)**
5-min	70.12 (0.12)	69.32 (0.13)	61.90 (0.12)**
*DBSCAN* + *RF*			
30-min	87.68 (0.09)	87.53 (0.10)	75.54 (0.14)**
20-min	87.98 (0.09)	87.66 (0.09)	75.91 (0.13)**
15-min	84.98 (0.11)	84.34 (0.12)	73.69 (0.13)**
10-min	85.29 (0.10)	84.85 (0.11)	74.64 (0.13)**
5-min	81.12 (0.12)	80.71 (0.12)	74.00 (0.10)**
*DBSCAN* + *MLP*			
30-min	80.07 (0.14)	80.29 (0.13)	70.50 (0.14)**
20-min	79.73 (0.13)	81.57 (0.12)	71.80 (0.14)**
15-min	78.81 (0.13)	78.75 (0.13)	70.23 (0.14)**
10-min	79.50 (0.12)	80.05 (0.12)	71.19 (0.12)**
5-min	76.26 (0.13)	76.49 (0.13)	68.90 (0.11)**
*K-means* + *LR*			
30-min	78.55 (0.13)	78.21 (0.11)	63.91 (0.15)**
20-min	78.95 (0.13)	78.59 (0.13)	65.51 (0.14)**
15-min	75.70 (0.13)	75.15 (0.13)	64.87 (0.13)**
10-min	76.00 (0.12)	75.11 (0.12)	65.10 (0.12)**
5-min	69.99 (0.13)	69.32 (0.13)	61.84 (0.11)**
*K-means* + *RF*			
30-min	87.66 (0.09)	87.53 (0.10)	75.37 (0.14)**
20-min	87.87 (0.09)	87.66 (0.09)	76.11 (0.13)**
15-min	84.71 (0.11)	84.34 (0.12)	73.86 (0.13)**
10-min	85.09 (0.10)	84.85 (0.11)	74.43 (0.13)**
5-min	81.16 (0.12)	80.71 (0.12)	73.88 (0.10)**
*K-means* + *MLP*			
30-min	79.76 (0.14)	80.29 (0.13)	70.41 (0.15)**
20-min	79.92 (0.13)	81.57 (0.12)	71.05 (0.13)**
15-min	78.68 (0.13)	78.75 (0.13)	70.34 (0.14)**
10-min	79.40 (0.12)	80.05 (0.12)	70.85 (0.12)**
5-min	75.80 (0.14)	76.49 (0.13)	68.75 (0.12)**
*DFI* + *LR*			
30-min	77.45 (0.12)	78.21 (0.11)	62.15 (0.13)**
20-min	78.38 (0.13)	78.59 (0.13)	64.57 (0.13)**
15-min	74.97 (0.14)	75.15 (0.13)	63.26 (0.13)**
10-min	74.98 (0.12)	75.11 (0.12)	63.82 (0.12)**
5-min	69.37 (0.13)	69.32 (0.13)	61.36 (0.10)**
*DFI* + *RF*			
30-min	86.8 (0.11)	87.53 (0.1)	75.63 (0.14)**
20-min	87.18 (0.1)	87.66 (0.09)	75.48 (0.13)**
15-min	83.8 (0.12)	84.34 (0.11)	73.44 (0.13)**
10-min	84.36 (0.11)	84.85 (0.1)	74.19 (0.13)**
5-min	80.78 (0.11)	80.71 (0.11)	72.82 (0.11)**
*DFI* + *MLP*			
30-min	79.90 (0.14)	80.29 (0.13)	69.94 (0.14)**
20-min	79.75 (0.14)	81.57 (0.12)	72.30 (0.13)**
15-min	78.57 (0.13)	78.75 (0.13)	70.46 (0.14)**
10-min	79.76 (0.12)	80.05 (0.12)	71.11 (0.12)**
5-min	75.93 (0.13)	76.49 (0.13)	68.32 (0.12)**

*DBSCAN* Density-Based Spatial Clustering of Applications with Noise, *LR* Logistic Regression, *RF* Random Forest, *MLP* Multilayer Perceptron, K-means - K-means Clustering, *DFI* Distance From Initial.

**p < 0.005 vs. the All-Features model using Wilcoxon Signed-Rank.

Another notable finding is that we observed a trend where larger, half-time intervals, 10-/15-/20-min, generally yielded higher predictive performance than 5-min, likely because longer windows more fully captured the duration of a smoking episode and compensated for variability in self-reported timestamps.

### Sensitivity analysis of possible COVID-19 emergency effects

Table 4 summarizes the results of the sensitivity analysis comparing prediction accuracy between two subgroups of study participants – during and after the COVID-19 emergency. Across all modeling configurations, the exclusion of temporal features also resulted in a statistically significant drop in Macro-F1 scores within the during-COVID group (*p* < 0.005), reinforcing the critical importance of temporal cues in predicting smoking events. For the post-COVID group, although only 22 out of 45 time-exclusion comparisons reached statistical significance (*p* < 0.05), many models still showed performance declines of over 10 percentage points, suggesting a strong, albeit underpowered, temporal signal. This partial loss of significance may be attributed to the smaller sample size of the post-COVID group (*n* = 9). In contrast, excluding location features led to only minor changes in performance across both groups, with no consistent or significant trends—further underscoring the robustness of temporal features over spatial ones in this predictive task.

**Table 4:** Summary of Sensitivity Analysis (Macro-F1 Score (unit: %))

Methods combinations between location representation & modeling	During Covid-19 Emergency Group			Post Covid-19 Emergency Group		
Half time interval	All features Mean (SD)	Location Excluded Mean (SD)	Time Excluded Mean (SD)	All features Mean (SD)	Location Excluded Mean (SD)	Time Excluded Mean (SD)
*DBSCAN* + *LR*						
30-min	79.24 (0.11)	78.19 (0.11)	61.91 (0.11)**	78.84 (0.15)	78.25 (0.13)	67.81 (0.18)*
20-min	78.54 (0.12)	78.34 (0.13)	63.44 (0.13)**	79.89 (0.15)	79.51 (0.15)	67.20 (0.14)*
15-min	76.44 (0.11)	75.65 (0.12)	64.22 (0.13)**	74.18 (0.18)	73.56 (0.18)	64.13 (0.15)*
10-min	75.62 (0.11)	75.26 (0.11)	63.83 (0.12)**	74.05 (0.16)	74.64 (0.16)	64.73 (0.13)*
5-min	71.29 (0.10)	70.67 (0.11)	62.73 (0.11)**	66.86 (0.16)	65.56 (0.18)	59.61 (0.14)*
*DBSCAN* + *RF*						
30-min	87.77 (0.09)	87.60 (0.10)	75.36 (0.14)**	87.37 (0.12)	87.29 (0.11)	76.19 (0.14)
20-min	88.18 (0.08)	87.69 (0.10)	76.14 (0.13)**	87.30 (0.10)	87.53 (0.09)	75.06 (0.13)*
15-min	86.72 (0.08)	86.24 (0.09)	75.18 (0.12)**	79.52 (0.16)	78.38 (0.17)	69.01 (0.16)
10-min	86.99 (0.08)	86.57 (0.08)	76.13 (0.12)**	79.96 (0.16)	79.45 (0.16)	69.98 (0.15)*
5-min	83.53 (0.10)	83.00 (0.10)	75.40 (0.11)**	74.40 (0.15)	74.36 (0.14)	70.10 (0.10)
*DBSCAN* + *MLP*						
30-min	80.20 (0.12)	80.02 (0.12)	69.73 (0.14)**	79.61 (0.19)	81.26 (0.17)	73.25 (0.15)
20-min	79.94 (0.12)	81.93 (0.11)	70.80 (0.14)**	78.98 (0.19)	80.30 (0.16)	75.39 (0.13)
15-min	80.23 (0.10)	80.38 (0.10)	70.51 (0.14)**	74.39 (0.20)	73.67 (0.20)	69.35 (0.15)
10-min	80.82 (0.10)	81.24 (0.09)	71.76 (0.12)**	75.36 (0.17)	76.35 (0.17)	69.40 (0.14)
5-min	78.72 (0.11)	78.67 (0.10)	69.35 (0.12)**	69.45 (0.17)	70.43 (0.17)	67.65 (0.10)
*K-means* + *LR*						
30-min	78.97 (0.12)	78.19 (0.11)	63.31 (0.14)**	77.03 (0.16)	78.25 (0.13)	66.03 (0.19)*
20-min	78.94 (0.12)	78.34 (0.13)	64.76 (0.13)**	78.99 (0.16)	79.51 (0.15)	68.17 (0.16)*
15-min	76.34 (0.12)	75.65 (0.12)	65.20 (0.13)**	73.70 (0.18)	73.56 (0.18)	63.84 (0.14)*
10-min	76.42 (0.11)	75.26 (0.11)	64.97 (0.12)**	74.69 (0.16)	74.64 (0.16)	65.50 (0.14)*
5-min	71.33 (0.11)	70.67 (0.11)	62.41 (0.11)**	66.29 (0.17)	65.56 (0.18)	60.27 (0.14)*
*K-means* + *RF*						
30-min	87.63 (0.09)	87.60 (0.10)	75.13 (0.14)**	87.77 (0.11)	87.29 (0.11)	76.26 (0.14)*
20-min	88.19 (0.09)	87.69 (0.10)	76.22 (0.13)**	86.73 (0.11)	87.53 (0.09)	75.71 (0.13)*
15-min	86.66 (0.08)	86.24 (0.09)	75.46 (0.12)**	78.63 (0.17)	78.38 (0.17)	68.86 (0.16)
10-min	86.77 (0.08)	86.57 (0.08)	76.00 (0.12)**	79.86 (0.16)	79.45 (0.16)	69.52 (0.15)*
5-min	83.71 (0.09)	83.00 (0.10)	75.27 (0.11)**	74.07 (0.15)	74.36 (0.14)	70.01 (0.10)
*K-means* + *MLP*						
30-min	79.77 (0.13)	80.02 (0.12)	69.25 (0.15)**	79.74 (0.20)	81.26 (0.17)	74.56 (0.14)
20-min	80.09 (0.12)	81.93 (0.11)	70.94 (0.13)**	79.29 (0.18)	80.30 (0.16)	71.45 (0.16)
15-min	79.79 (0.11)	80.38 (0.10)	70.47 (0.14)**	75.19 (0.18)	73.67 (0.20)	69.92 (0.15)
10-min	80.82 (0.09)	81.24 (0.09)	71.41 (0.12)**	74.94 (0.17)	76.35 (0.17)	69.09 (0.14)
5-min	78.00 (0.12)	78.67 (0.10)	69.67 (0.12)**	69.69 (0.17)	70.43 (0.17)	66.18 (0.11)
*DFI* + *LR*						
30-min	77.37 (0.12)	78.19 (0.11)	60.81 (0.11)**	77.73 (0.14)	78.25 (0.13)	66.93 (0.19)*
20-min	78.11 (0.12)	78.34 (0.13)	63.36 (0.13)**	79.37 (0.15)	79.51 (0.15)	68.89 (0.15)*
15-min	75.37 (0.12)	75.65 (0.12)	63.16 (0.13)**	73.71 (0.18)	73.56 (0.18)	63.57 (0.15)*
10-min	75.23 (0.11)	75.26 (0.11)	63.09 (0.11)**	74.21 (0.17)	74.64 (0.16)	66.09 (0.13)*
5-min	70.48 (0.11)	70.67 (0.11)	61.23 (0.10)**	66.28 (0.17)	65.56 (0.18)	61.72 (0.12)
*DFI* + *RF*						
30-min	86.71 (0.11)	87.60 (0.10)	75.40 (0.14)**	87.13 (0.11)	87.29 (0.11)	76.45 (0.14)
20-min	87.39 (0.10)	87.69 (0.10)	75.63 (0.13)**	86.45 (0.11)	87.53 (0.09)	74.92 (0.14)*
15-min	85.70 (0.09)	86.24 (0.09)	74.88 (0.12)**	77.85 (0.18)	78.38 (0.17)	68.93 (0.16)*
10-min	85.96 (0.08)	86.57 (0.08)	75.76 (0.12)**	79.38 (0.16)	79.45 (0.16)	69.27 (0.15)*
5-min	83.01 (0.10)	83.00 (0.10)	74.09 (0.11)**	74.56 (0.13)	74.36 (0.14)	69.27 (0.10)
*DFI* + *MLP*						
30-min	79.93 (0.12)	80.02 (0.12)	68.73 (0.14)**	79.81 (0.20)	81.26 (0.17)	74.25 (0.15)
20-min	79.71 (0.12)	81.93 (0.11)	71.32 (0.13)**	79.91 (0.19)	80.30 (0.16)	75.79 (0.12)
15-min	79.82 (0.11)	80.38 (0.10)	70.99 (0.14)**	74.65 (0.20)	73.67 (0.20)	68.81 (0.15)
10-min	81.16 (0.10)	81.24 (0.09)	71.51 (0.12)**	75.38 (0.18)	76.35 (0.17)	69.86 (0.14)
5-min	78.52 (0.11)	78.67 (0.10)	69.12 (0.12)**	68.72 (0.18)	70.43 (0.17)	66.08 (0.11)

*DBSCAN* Density-Based Spatial Clustering of Applications with Noise, *LR* Logistic Regression, *RF* Random Forest, *MLP* Multilayer Perceptron, *K-means* K-means Clustering, *DFI* Distance From Initial.

*p < 0.05 vs. the All-Features model using the Wilcoxon Signed-Rank.

**p < 0.005 vs. the All-Features model using the Wilcoxon Signed-Rank.

### Associations between location and time of day

The mixed-effects modeling analysis for examining possible explanations for the prediction results revealed a high number of significant associations between each temporal feature (i.e., the 96 quarter-hour and 48 half-hour intervals in a 24-h period) and location clusters. The support value for each statistically significant association ranged from 47.39% to 81.90% depending on smoking status and time granularity (see Supplementary Tables 3–6).

## Discussion

Our analysis suggests that the removal of location features from a model that uses time and location to predict smoking events results in a very small decrease in the predictive power of the model. Conversely, excluding time-related features, such as the time of day and day of the week, resulted in a significant drop in predictive performance, as evidenced by a substantial decrease in the Macro-F1 scores. Importantly, this conclusion held across all machine learning (ML) modeling approaches employed (LR, RF, MLP) and across different spatial feature representations, including DBSCAN, K-means, and DFI. Moreover, the pattern also remained stable when stratifying participants into subgroups based on their study participation period—whether during or after the COVID-19 emergency. The consistency in these findings suggest that temporal factors are more predictive than location for smoking behavior, perhaps because they align with habitual routines and patterns that are deeply ingrained in the daily lives of people who smoke thereby making location information redundant with temporal information. These results have implications for the design of JITAIs to assist with the smoking cessation attempt and could greatly simplify the development of JITAIs as well as alleviate privacy concerns.

The results reported in this study also indicate that, within the context of this study, in which the data was collected in naturalistic settings, location alone was less able than time to predict smoking events and did not significantly improve the ability to predict smoking when added to time. A prior analysis that had been performed on the same raw dataset as was used in the current study (but with different pre-processing) found that location^26^ could provide information to models that attempt to detect smoking events based on physiological markers such as heart rate and heart rate variability (HRV). However, that prior analysis also noted that while GPS data improved accuracy in individual-level models, its predictive power significantly diminished when generalizing across participants, especially in leave-one-out cross-validation (LOOCV). This suggests a high dependency on personal context, meaning location features may capture only a few certain individual smoking patterns but lack consistency across broader populations (HRV prevails over location among them) based on that study. Our analysis, by focusing specifically on location and temporal cues independently, revealed that time features had a more robust and generalizable predictive power, while location alone was not consistently effective. This divergence highlights that location’s value may be context-dependent and potentially overfitted in individual-level training, which aligns with our findings that temporal patterns offer more reliable predictors across different individuals.

Smokers may be more influenced by the time of day rather than specific locations perhaps due to the flexibility and mobility of their daily activities. An alternative explanation may be that location tends to be redundant with time in the context of smoking events - i.e., people are creatures of habit and tend to smoke in the same locations at the same times. The range of the support value for each statistically significant association between location and time of day when smoking was reported by study participants may indicate that temporal patterns often encode the same predictive information as location, reducing the independent contribution of location features when time is already considered. The fact that not all of the time intervals are significantly associated with location during reported smoking events may also suggest that some location information is not completely redundant with time but is likely not relevant to smoking events.

The findings of the current study are particularly important in light of other studies that have investigated the use of geofencing and geolocation to tailor supportive messages to individual participants during a quit attempt. For example, Naughton et al. found that supportive messages triggered by geofences on smartphones were reported by study participants to be helpful during their quit attempt^35^. However, Naughton et al.’s findings are based on post-hoc self-reported information and semistructured interviews, as their study was not designed to determine the relative contribution of various potential predictors of smoking events. In contrast, the findings of our present study advocate for a greater emphasis on temporal cues when developing predictive models and interventions for smoking cessation, as they appear to offer more consistent and reliable indicators of smoking behavior compared to spatial features. Nonetheless, we acknowledge that qualitative methods, such as interviews or follow-up discussions, as used in Naughton et al.’s study, can provide valuable contextual insights into participant behaviors that quantitative analyses alone may not fully uncover. Future research may benefit from combining both approaches to better interpret the behavioral drivers behind predictive features like time of day.

The fact that the Random Forest and the MLP models outperformed the linear logistic regression model may indicate that there is a non-linear component in the relationship between time and smoking events. The best performance (F1 score of 87.98) was achieved with an RF model trained on features extracted from 20-min half-time windows and DBSCAN used for location clustering with the RF model that excluded location clustering performing only less than 1 point worse (F1-score of 87.66). However, the RF model was still highly predictive even with 5 min windows in both conditions (with and without location), which makes this approach suitable for a wider range of smoking frequencies (e.g., heavy smokers).

In summary, the major practical implication of our findings is that excluding geolocation from features used to model smoking behavior for JITAIs does not significantly reduce the accuracy of predicting smoking events but has the advantage of reducing the complexity of monitoring the behavior, decreasing concerns with privacy and decreasing the impact of such monitoring on the smartphone’s battery life.

While this analysis provides valuable insights into the role of temporal and spatial features in predicting smoking events, the results of this study should be interpreted in light of several limitations. First, the study relied on self-reported smoking events, which may be subject to recall bias or inaccuracies. Although participants were instructed to log their smoking instances promptly, delays or missed entries could have impacted the accuracy of the data. Potentially, by incorporating biochemical verification and passive sensing, researchers and clinicians can greatly improve the accuracy of smoking data in naturalistic settings. For example, biochemical tests, such as exhaled carbon monoxide concentrations or cotinine concentrations measured in urine or saliva can validate self-reported abstinence, strengthening confidence in outcomes^30,41^. On the other hand, passive sensors such as smart lighters, and smartphones can provide ongoing, real-world monitoring that captures granular smoking behaviors that diaries might miss^42^. Together, these approaches help overcome recall errors and intentional misreporting, mitigating the limitations of self-reported data.

Second, the GPS data used to capture spatial features was relatively coarse and may not have accurately reflected the specific environments or contexts in which smoking occurred. For indoor-outdoor distinctions as an example, the resolution of GPS data typically ranges from 5 to 30 m outdoors, but often significantly worsens while indoors or near large structures due to satellite signal blockage or reflection, making it much more challenging for precise indoor-outdoor distinctions using GPS alone^43,44^. These limitations in spatial granularity could have led to miscategorized labels of spatial clusters. Alternative or complementary location-sensing technologies, such as Wi-Fi fingerprinting^45–47^, Bluetooth beacons^48,49^, or barometric sensors^50^, could provide more accurate spatial resolution and finer-grained indoor-outdoor classification^43^; however, such methods would also make location sensing more complicated, expensive and therefore less feasible.

Third, the study’s focus on temporal and spatial features did not account for other potential predictors of smoking behavior, such as social interactions, stress levels, or access to cigarettes, which may also play a crucial role in triggering smoking events. Moreover, while temporal features demonstrated strong predictive utility at the population level, individual-specific triggers and contextual nuances—such as personalized routines, emotional states, or social environments—may still be overlooked in this aggregated analysis. Future studies could benefit from incorporating a broader range of contextual and psychological variables and leveraging personalized or adaptive modeling approaches to capture individual-level variation in smoking behavior.

Lastly, our study’s generalizability is constrained by its relatively modest number of participants (*n* = 38 participants), although the relatively long duration of the study somewhat compensated for that limitation by resulting in 20,304 samples in total. A larger, more diverse sample of participants might uncover subtler spatial patterns and allow better subgroup analyses, which would strengthen confidence in the broader applicability of these findings. Future studies incorporating more participants across diverse contexts (e.g., urban versus rural settings, varying socioeconomic backgrounds) would help confirm the generalizability and robustness of temporal predictors relative to spatial information. These limitations highlight the need for future research to refine the methods and data used to study smoking behavior, potentially leading to more effective predictive models and intervention strategies.

## Methods

### Dataset

The dataset utilized in this study originates from an Institutional Review Board-approved research project conducted at the University of Minnesota. Participants were recruited nationwide in the United States, with all study procedures designed to be conducted remotely due to Coronavirus Disease 2019 (COVID-19) restrictions present at the time of study initiation. Eligibility criteria included that participants were at least 21 years old and reported smoking an average of at least 10 cigarettes per day over the past year. During the study, participants were asked to wear multiple wearable sensors continuously while going about their daily routines. Participants used a custom-built smartphone application, PhysiAware™ (iOS and Android), to collect data from the sensors and to log on the app each smoking event including where the smoking event took place (e.g., home, car, work, bar/restaurant, etc.) and the perceived reason for each smoking instance (e.g., stress, boredom, habit, etc.). Data collected included a continuous stream of GPS coordinates adjusted using a random offset value while preserving the relative distances between the coordinates within each participant’s subset of the data to preserve privacy. Recording of GPS coordinates was triggered by movement greater than 100 feet as well as the exact time the smoking event was logged. Participants were asked to quit smoking approximately half-way through the 14 day observation period but were allowed to postpone the quit date for up to 1 week. Therefore some participants reported up to 2 weeks of pre-quit data (and up to 3 weeks of data in total). The smoking reports and location data occurring between the first date of data collection and the start of the actual quit date were used for the analyses described in this paper. By this means, we excluded post-quit data since smoking patterns would likely change when smokers are actively resisting smoking during the period when they would normally smoke. This doesn’t undermine the premise of the current study since these time periods are likely when the participants are at high risk of lapsing (even if they don’t actually lapse) and therefore need support from the JITAI. This study was approved by the Institutional Review Board (IRB) of the University of Minnesota (approval number: STUDY00012088) and adhered to the guidelines outlined in the Declaration of Helsinki, ensuring compliance with ethical standards for research involving human participants. All participants provided written informed consent prior to participating in the research. Participants were fully briefed on the purpose and procedures of the study and provided their consent voluntarily. For this study, all data collection was conducted remotely, and participants had the opportunity to withdraw from the study at any time without penalty.

A potential confounder for determining the contribution of geolocation features is that the PhysiAware study began during the COVID-19 national emergency which limited mobility and changed movement patterns of individuals. While our data indicates that study participants were not confined to one place (mean number of geolocation clusters as 3.22 (SD 3.20)); we wanted to perform a sensitivity analysis comparing the smoking event prediction results between the group of participants that enrolled in the study during the COVID-19 emergency and the smaller group that enrolled after the federal Public Health Emergency declaration expired on May 11, 2023.

### Data harmonization

**Fig. 1:**
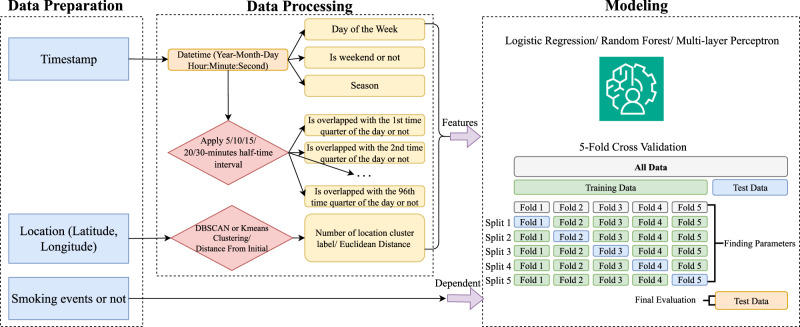
Overview of data harmonization. This figure illustrates the complete study workflow, starting from raw data collection through predictive modeling. Participants reported smoking events using a mobile app, which also passively collected GPS coordinates during periods of significant movement. Each event’s timestamp was first converted into a datetime format, from which several temporal features were derived, including day of the week, whether the day was a weekend, season of the year, and 96 quarter-hour interval binary features per day. GPS coordinates (latitude and longitude) were processed using unsupervised clustering (DBSCAN, K-means) or converted to Euclidean distances from the initial recorded location (DFI) to extract spatial patterns. Using half-time-interval windows (e.g., ±5-, 10-, 15-, 20-, or 30-minute), each time point was labeled as smoking or non-smoking, transforming the task into a binary classification problem. Three modeling approaches—logistic regression, random forest, and multilayer perceptron—were trained and evaluated using 5-fold cross-validation. Each participant’s prediction results were summarized by Macro-F1 scores, and statistical comparisons (Wilcoxon signed-rank tests) were conducted to evaluate the predictive contribution of temporal vs. spatial features. A sensitivity analysis also performed under the same pipeline but comparing the smoking event prediction results between the group of participants that enrolled in the study during the COVID-19 emergency and the smaller group that enrolled after the federal Public Health Emergency declaration expired on May 11, 2023.

Figure 1 shows an overview of the data harmonization process used in this study, which involves several key steps to prepare data for analysis. Initially, raw timestamps and location data (latitude and longitude) are collected alongside smoking event labels. These timestamps are converted into a detailed datetime format, from which temporal features such as day of the week, whether the day is a weekend, and seasonal information are extracted. The study applies a 5/10/15/20/30-min half-time interval (see section below) to the timestamps to approximate the duration of smoking events and identifies overlaps with specific quarter-hour periods of the day. Concurrently, location data is processed using three methods: DBSCAN and K-means clustering to assign location cluster labels capturing significant spatial patterns, and a simple euclidean distance from the initial (anchor) location. These temporal and spatial features are then used to train three ML models including logistic regression, random forest, and multi-layer perceptron. The models’ performance was validated with a 5-fold cross-validation approach for improved robustness and reliability in predicting smoking events.

To approximate the actual duration of each smoking event, we applied a half-time interval around the self-reported smoking timestamp. Although smoking events are reported as single time points, they generally span several minutes. Additionally, timestamps may be imprecise due to recall limitations in naturalistic settings. To account for these factors, we defined half-time intervals extending forward and backward from each reported time, testing 5-, 10- and 15-, 20-, and 30-minute intervals to approximate typical smoking durations and capture overlapping temporal patterns more accurately in our analysis. Thus, each smoking event for the purposes of predictive modeling is treated as a time interval rather than a discrete time point.

Each record used to train an ML model represents a combination of the user’s smoking report and continuously collected GPS data, matched via timestamps. If a user reported smoking, the period from the reported time plus and minus a chosen half-time interval (we experimented with all 5-, 10-, 15-, 20- and 30-min intervals) was assumed to be the smoking duration. GPS records during this interval were labeled as smoking events for predictive modeling purposes. Time ranges (and the GPS positions therein) outside these intervals labeled as smoking, were classified as non-smoking events. This approach enabled us to frame the research as a binary classification problem.

Temporal features were derived from the raw timestamps associated with each recorded event to capture the temporal patterns of smoking behavior. The first step involved converting the raw timestamps into a structured datetime format, which includes the year, month, day, hour, minute, and second. From this detailed datetime information, several key temporal features were extracted:

1. Day of the Week: Each event was labeled with the corresponding day of the week.
2. Weekend Indicator: A binary feature was created to distinguish between events occurring on weekends versus weekdays since smoking behavior may differ on weekends due to changes in daily routines.
3. Season: The events were categorized into one of four seasons (spring, summer, fall, winter) based on the date.
4. Quarter-Hour Intervals: To capture daily temporal patterns, we divided each day into 96 Quarter-Hour Intervals. Each interval is labeled as either 1 or 0, based on whether it overlaps with a smoking event. Using the predefined half-time intervals (5-, 10-, 15-, 20- or 30-minute), the labels for Quarter-Hour Intervals reflect the presence or absence of smoking events around each timestamp. For example, suppose a smoking event is reported at 9:10 a.m. with a 15-min half-time interval. This event would extend from 8:55 to 9:25 a.m., covering the 8:45-9:00, 9:00-9:15, and 9:15-9:30 Quarter-Hour Intervals, labeling each as 1. Other intervals, such as 9:30-9:45, would be labeled 0 until the next reported smoking event.

These temporal profiles were then used in the modeling process to assess their contribution to predicting smoking events, providing insights into how time-related factors influence smoking behavior.

### Machine learning

To evaluate the predictive utility of spatial features, we extracted location-based variables from GPS coordinates (latitude and longitude) recorded during each participant’s pre-quit study period. Our primary spatial representation used Density-Based Spatial Clustering of Applications with Noise (DBSCAN)^51^, a clustering algorithm particularly well-suited for spatial data due to its ability to identify arbitrarily shaped clusters and distinguish noise without requiring a pre-specified number of clusters. DBSCAN defines clusters as dense regions separated by areas of low point density, making it robust in detecting meaningful location patterns even when the data includes outliers or irregular sampling. This characteristic is advantageous in naturalistic behavioral data, where participants may log events at inconsistent rates and locations may not follow geometric regularities^34,52–55^. Moreover, DBSCAN’s ability to manage spatial noise is consistent with methods used in prior behavioral health studies, such as Maxhuni et al.’s work on geolocation-driven stress prediction^54^ and Yang et al.’s application for visualizing spatial tobacco data patterns^55^.

DBSCAN was used to cluster each participant’s location history, yielding participant-specific location labels. This method helped identify personalized smoking hotspots, which could then be incorporated into predictive modeling. DBSCAN was chosen over traditional clustering algorithms for its adaptability to varying data densities and its minimal assumptions about location distribution. To assess the robustness of spatial feature extraction and address potential concerns about model sensitivity to clustering methods, we conducted two ablation studies using alternative location representations.

The first ablation study replaced DBSCAN with K-means clustering^56^, a widely-used algorithm that partitions data into a pre-specified number of clusters by minimizing intra-cluster variance. Unlike DBSCAN, K-means assumes spherical, evenly sized clusters and is sensitive to outliers, but it offers computational simplicity and has been applied in various health informatics applications involving geo-spatial patterns^57–59^. For each participant, K-means clustering was applied independently using an automatically selected number of clusters using the silhouette score^60^. The results showed that predictive performance remained largely consistent with those obtained using DBSCAN, suggesting that while the clustering method has some impact, temporal features still dominate overall performance.

The second ablation study used a simpler representation of spatial information: the Euclidean Distance from Initial location (DFI). For each event, we computed the straight-line distance from the participant’s initial recorded location, serving as a proxy for mobility. This approach offers a continuous, directly computable, and interpretable measure of spatial displacement without relying on clustering algorithms. Some prior work has shown that spatial mobility features such as displacement and travel radius can meaningfully relate to health-related behaviors^61–63^. In our case, DFI was used as the spatial feature resulting in comparable performance to clustering-based approaches.

Logistic regression (LR) is a widely used method for binary classification tasks, where the goal is to predict the probability that a given input belongs to one of two categories. Unlike linear regression, which predicts continuous outcomes, LR is designed to estimate the likelihood of a binary event occurring. This is done by modeling the relationship between the dependent variable (in our case, whether a smoking event occurs or not) and independent variables (i.e., features, the given time and location conditions)^64,65^.

A strength of LR lies in its ability to provide insight into the contribution of each feature to the prediction task. By assigning weights to the features based on their influence on the predicted outcome, LR allows for a clear comparison of the predictive power of different feature sets^66,67^. This capability is particularly useful in studies like ours, where the objective is to assess the relative importance of various factors, such as the time of day, location, and other contextual information, in predicting smoking events.

To evaluate the impact of specific features, we can train multiple LR models, each with a different combination of features. By systematically excluding or including targeted features - such as time of day interval identifier or location cluster label - we can observe changes in model performance metrics (i.e., prediction accuracy, precision, recall, and the Macro F1-score). Comparing these metrics across models enables us to quantify how much each feature contributes to the overall predictive accuracy. This method allows us to determine whether certain features, such as time of day or location, significantly enhance the model’s ability to predict smoking events.

Random Forest (RF) is an ensemble learning method that constructs multiple decision trees by randomly sampling data points and features, combining their predictions to improve model accuracy and generalizability^68^. Unlike LR, RF can naturally capture complex non-linear relationships between variables, which makes it advantageous in behavioral prediction contexts where relationships among features often deviate from linearity^69–71^. Additionally, RF models are relatively robust and effective even with moderate sample sizes and imbalanced datasets, providing stable estimates and consistent predictive performance^72–74^. These characteristics have led to some applications of RF in digital health studies, including mobile-sensor-based smoking behavior prediction and JITAI development^37,75^.

Multilayer perceptron (MLP) is the feedforward neural network (NN)^76^ consisting of fully connected neurons with nonlinear activation functions, organized in layers, notable for being able to distinguish data that is not linearly separable^77,78^. This capacity allows MLPs to capture intricate patterns that linear models like LR might overlook^79,80^. Specifically, we employed a MLP with two hidden layers, comprising 300 neurons in the first layer and 150 in the second. This architecture balances model complexity and computational efficiency, which is particularly important given our sample size. The Rectified Linear Unit (ReLU) activation function^81^ was applied to each neuron to introduce non-linearity. Then for optimization, we utilized the Adam optimizer^82^, known for its adaptive learning rates and computational efficiency, facilitating faster convergence compared to standard stochastic gradient descent.

K-fold cross-validation is a robust technique that offers some protection from spurious findings and is widely used to evaluate machine learning models. In this approach, the dataset is divided into k equal parts (*k* = 5 in our case), or “folds.” The model is trained on four of these folds and tested on the remaining one, and this process is repeated k times, each time with a different fold as the test set. The results from all k iterations are averaged to provide an assessment of the model’s performance. This method helps mitigate the risk of overfitting by ensuring that the model is tested against different subsets of the data, making the evaluation more reliable, repeatable and reflective of how the model may perform on unseen data^83^.

### Statistical methods

The Macro-F1 score is the main evaluation metric chosen for this study particularly due to the slightly imbalanced nature of the dataset. As summarized in Table 1, on average, each participant generated 487.37 GPS records, but only 46.95 smoking events reported in the pre-quit study period used in the current study. This imbalance necessitates a metric that equally considers the performance across both classes (smoking and non-smoking events) rather than being dominated by the majority class. The Macro-F1 score calculates the harmonic mean between precision and recall for each class independently and then averages them, ensuring that the model’s performance is fairly evaluated even when the class distribution is uneven^84,85^. By using the Macro-F1 score, we aim to assess the model’s ability to correctly predict smoking events (the minority class) without being biased by the more frequent non-smoking events.

To evaluate the significance of the differences in Macro-F1 scores when excluding specific features (such as location or time), the Wilcoxon signed-rank test is employed. This non-parametric test is particularly suitable for our study, as it does not assume a normal distribution of differences between paired samples, making it suitable for participant-wise comparisons^86^. For each participant, we calculate the Macro-F1 score for the model that includes all features and compare it to the scores of models from which either location or time features are excluded. By applying the Wilcoxon signed-rank test, we assess whether the observed changes in Macro-F1 scores are statistically significant, thereby determining whether the exclusion of location or time information substantially impacts the model’s predictive power^87^. A Student’s T-test for independent samples was used to test for differences in participant characteristics including age, duration of observations and number of reported smoking events.

To investigate the correlation between temporal features and location specifically, we employed linear mixed-effects models (LMMs) to account for the hierarchical structure of the data and the inherent correlations within observations grouped by individuals^88–90^. Unlike standard linear models that provide population-averaged estimates, mixed-effects models allow for subject-specific effects by including random intercepts or slopes for each grouping factor (e.g., the person’s study id). This approach partitions the overall variation in the dependent variable into components attributable to different hierarchical levels, such as between-individual and within-individual variation.

For two-fold effects, the fixed effects in the model represent the population-level effects of temporal features, such as quarter time of a day or half-hour time of a day, on location clusters. The random effects capture individual-specific variation, enabling the model to accommodate inter-individual heterogeneity while preserving interpretability. This combination of fixed and random effects makes LMMs particularly suitable for analyzing complex, longitudinal, or nested datasets where observations are not independent.

**Fig. 2:**
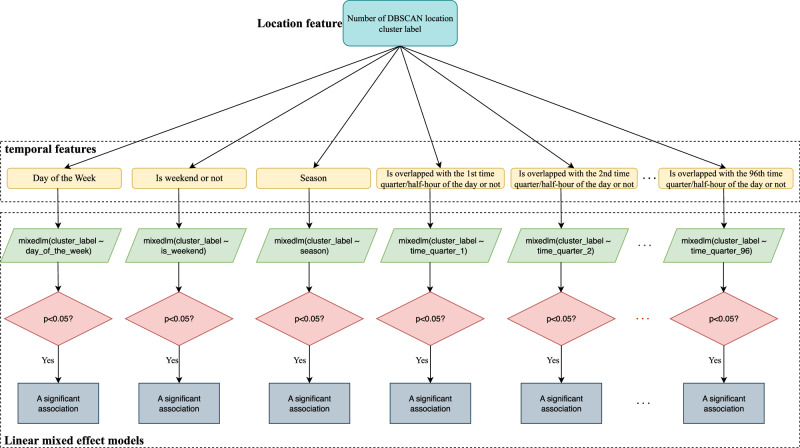
Workflow for applying linear mixed-effects models. This figure presents the modeling framework used to assess correlations between temporal features and spatial cluster labels across individual participants using linear mixed-effects models (LMMs). For each temporal feature—such as specific quarter-hour intervals, day of the week, seasonality, and weekend/weekday status—a mixed-effects model was constructed to predict location cluster labels while accounting for random intercepts at the participant level. This enabled estimation of fixed effects of time-related variables while controlling for repeated measures within individuals. The visualization highlights the statistical testing and coefficient extraction steps that follow model fitting. These results helped determine the extent to which temporal features alone could explain spatial variation, providing insight into whether location offers unique predictive value beyond what is already captured by temporal patterns. This analysis contributes to the conclusion that temporal features may subsume much of the information contained in spatial data, especially in the context of habitual smoking behavior.

As shown in Fig. 2, we fitted LMMs with each temporal feature iteratively and in various combinations (i.e., each feature treated separately for each participant resulting in $N$ (participants) $\times M$ (temporal features) models fitted), as predictors and assessed their associations with location clusters while grouping observations by each participant. The cases we chose to examine with LMMs included DBSCAN location clusters of smoking events with quarter-hour intervals and DBSCAN location clusters of smoking events with half-hour intervals. This allowed us to iteratively evaluate the significance of each temporal feature in predicting location-specific smoking behaviors while accounting for individual variability.

A statistical inference result was considered statistically significant if the probability of observing it by chance was less than 5% when the null hypothesis was true (i.e., alpha (ɑ) ≤ 0.05).

## Supplementary information

Supplementary information

## Data Availability

The data that support the findings of this study are available from the University of Minnesota, but restrictions apply to public availability of these data due to their protected nature and University of Minnesota data governance policies. Deidentified data are available from the authors upon reasonable request subject to institutional approvals. To facilitate reproducibility, all analysis code is provided along with a synthetic dataset that mimics the structure and features of the original data. This synthetic dataset and associated code are publicly accessible via our GitHub repository at https://github.com/UMN-RXInformatics/PhysiAware.

## Abbreviations

GPS: Global Positioning System
DBSCAN: Density-Based Spatial Clustering of Applications with Noise
K-means: K-means Clustering
DFI: Distance from Initial Geo-location
LR: Logistic Regression
RF: Random Forest
MLP: Multilayer Perceptron
ReLU: Rectified Linear Unit
SD: Standard Deviation
COPD: Chronic Obstructive Pulmonary Disease
JITAIs: Just-in-time Adaptive Interventions
COVID-19: Coronavirus Disease 2019
LOOCV: Leave-one-out Cross-validation
IRB: Institutional Review Board
HRV: Heart Rate Variability
ML: Machine Learning

## Code availability

The code used to preprocess data, extract features, and train the machine learning models (logistic regression, random forest, and multilayer perceptron) is available at our GitHub repository: https://github.com/UMN-RXInformatics/PhysiAware. The repository includes a synthetic dataset that replicates the structure and key properties of the original data to support reproducibility. Code was developed and executed using Python (version 3.8), with necessary packages including datetime, scikit-learn, pandas, numpy, and statsmodels. Key parameters such as DBSCAN (eps=0.01, min_samples=20), the architecture of the MLP (two hidden layers of 300 and 150 nodes with ReLU activation and Adam optimizer), and other models and settings are documented in the repository.
