# Supplementary material for: Relative importance of temporal and location features in predicting smoking events
Source: NPJ Digit Med. 2025 Jul 5;8:409. doi: 10.1038/s41746-025-01799-5 (PMC12227676; doi:10.1038/s41746-025-01799-5)
Supplement: Supplementary file 1 — Supplementary information [file 41746_2025_1799_MOESM1_ESM.pdf]

Supplementary Table 1 Independent T-test for the continuous variables: Age, Duration of data collection (days), Pre-quit study period's GPS positions, Smoking events, GPS data samples labeled as smoking, and DBSCAN geolocation clusters

Test Variable Name	T-statistic	P-value	Mean Difference	95% Confidence Interval	
Age	0.91	0.37	2.92	-3.58	9.43
Duration of data collection (days)	-0.13	0.9	-0.17	-2.16	1.82
**Timeframe: Pre-quit study period (up to 2 weeks)**					
GPS positions	0.90	0.38	313.22	-114.45	740.89
Smoking events	-0.72	0.48	-9.68	-35.08	15.73
All GPS data samples	0.86	0.39	298.40	-124.43	721.24
GPS data samples labeled as smoking	1.12	0.27	82.42	-16.13	180.96
DBSCAN geolocation clusters	-1.00	0.32	-0.81	-2.46	0.85

All the p-values here are greater than 0.1, indicating there were no statistically significant differences between the during–Covid-19 emergency group and the post-Covid-19 emergency group for these continuous variables.

Supplementary Table 2 Wilcoxon Signed-Rank Test Results Comparing the Macro-F1 Score Difference of All Features Model with of Location-Excluded and of Time-Excluded Models, a combination of DBSCAN and LR is applied

Half time interval	N	Full vs. Location-Excluded Models				Full vs. and Time-Excluded Models			
		test statistic	p-value	95% CI		test statistic	p-value	95% CI	
30 minutes	32	128.0	0.09	0	0.25	1.0	9.31E-10	0	2.63E-09
20 minutes	32	183.0	0.46	0	1	4.0	3.26E-09	0	9.20E-09
15 minutes	33	212.0	0.48	0	1	11.0	1.28E-08	0	3.61E-08
10 minutes	33	202.0	0.74	0	1	15.0	3.22E-06	0	9.09E-06

5 minutes	34	179.0	0.18	0	0.50	14.0	2.94E-06	0	8.30E-06

Supplementary Table 3 Mixed-Effects Model Results: Association Between Temporal Features and DBSCAN Location Clustering Labels, From the Smoking Events with Quarter-Hour Intervals

Variable	Support	Coef.	Std.Err.	z	P>|z|	0.025	0.975
day_of_week	4608	1.69	0.63	2.684	0.007*	0.456	2.924
is_weekend	1301	2.796	0.313	8.932	0*	2.182	3.41
season	4352	-6.424	12.939	-0.497	0.62	-31.784	18.935
time_quarter_0	43	5.824	1.439	4.046	0*	3.003	8.646
time_quarter_1	42	4.073	1.458	2.794	0.005*	1.216	6.93
time_quarter_2	30	3.499	1.718	2.037	0.042*	0.132	6.865
time_quarter_3	20	2.523	2.107	1.198	0.231	-1.606	6.652
time_quarter_4	25	1.622	1.887	0.86	0.39	-2.075	5.32
time_quarter_5	22	1.528	2.005	0.762	0.446	-2.402	5.457
time_quarter_6	17	1.355	2.281	0.594	0.553	-3.116	5.826
time_quarter_7	10	5.841	2.968	1.968	0.049*	0.024	11.658
time_quarter_8	8	7.279	3.32	2.192	0.028*	0.771	13.786
time_quarter_9	14	5.133	2.511	2.044	0.041*	0.212	10.054
time_quarter_10	29	4.115	1.747	2.356	0.018*	0.692	7.538
time_quarter_11	38	4.242	1.533	2.768	0.006*	1.238	7.246
time_quarter_12	35	5.364	1.596	3.361	0.001*	2.236	8.491

Variable	Support	Coef.	Std.Err.	z	P>|z|	0.025	0.975
time_quarter_13	20	4.191	2.097	1.998	0.046*	0.081	8.302
time_quarter_14	12	4.619	2.704	1.709	0.088	-0.68	9.919
time_quarter_15	7	2.633	3.544	0.743	0.458	-4.313	9.579
time_quarter_16	3	5.909	5.418	1.091	0.275	-4.71	16.528
time_quarter_17	1	5.869	9.353	0.627	0.53	-12.463	24.2
time_quarter_18	0	NA	NA	NA	NA	NA	NA
time_quarter_19	19	9.391	2.161	4.345	0*	5.154	13.627
time_quarter_20	39	8.094	1.514	5.345	0*	5.126	11.061
time_quarter_21	56	7.785	1.27	6.13	0*	5.296	10.274
time_quarter_22	55	6.919	1.278	5.416	0*	4.415	9.423
time_quarter_23	137	0.482	0.827	0.583	0.56	-1.139	2.103
time_quarter_24	285	-0.974	0.669	-1.457	0.145	-2.285	0.336
time_quarter_25	321	-0.817	0.623	-1.311	0.19	-2.039	0.404
time_quarter_26	278	0.758	0.643	1.18	0.238	-0.502	2.019
time_quarter_27	175	2.731	0.727	3.754	0*	1.305	4.157
time_quarter_28	156	2.799	0.769	3.641	0*	1.292	4.306
time_quarter_29	125	2.297	0.858	2.677	0.007*	0.615	3.978
time_quarter_30	90	2.073	1.017	2.037	0.042*	0.079	4.067
time_quarter_31	106	1.319	0.931	1.416	0.157	-0.507	3.144

Variable	Support	Coef.	Std.Err.	z	P>|z|	0.025	0.975
time_quarter_32	126	2.321	0.854	2.719	0.007*	0.648	3.994
time_quarter_33	161	1.25	0.763	1.639	0.101	-0.245	2.746
time_quarter_34	204	1.448	0.685	2.113	0.035*	0.105	2.79
time_quarter_35	216	0.981	0.676	1.451	0.147	-0.344	2.306
time_quarter_36	187	1.631	0.715	2.283	0.022*	0.231	3.032
time_quarter_37	155	1.045	0.784	1.333	0.182	-0.491	2.582
time_quarter_38	128	0.8	0.867	0.923	0.356	-0.899	2.498
time_quarter_39	139	0.723	0.833	0.869	0.385	-0.908	2.355
time_quarter_40	103	0.988	0.938	1.053	0.292	-0.851	2.827
time_quarter_41	149	1.495	0.797	1.876	0.061	-0.067	3.056
time_quarter_42	249	-10.648	0.609	-17.475	0*	-11.842	-9.454
time_quarter_43	271	-10.274	0.585	-17.55	0*	-11.421	-9.126
time_quarter_44	228	-12.64	0.631	-20.039	0*	-13.876	-11.403
time_quarter_45	134	0.252	0.828	0.304	0.761	-1.37	1.874
time_quarter_46	170	2.471	0.736	3.358	0.001*	1.029	3.913
time_quarter_47	225	2.687	0.646	4.16	0*	1.421	3.953
time_quarter_48	219	2.491	0.659	3.782	0*	1.2	3.782
time_quarter_49	192	-1.255	0.7	-1.794	0.073	-2.627	0.116
time_quarter_50	125	-3.306	0.855	-3.864	0*	-4.982	-1.629

Variable	Support	Coef.	Std.Err.	z	P>|z|	0.025	0.975
time_quarter_51	162	-5.332	0.759	-7.022	0*	-6.82	-3.844
time_quarter_52	209	-0.095	0.677	-0.141	0.888	-1.423	1.232
time_quarter_53	237	0.359	0.644	0.558	0.577	-0.903	1.622
time_quarter_54	222	3.062	0.658	4.656	0*	1.773	4.351
time_quarter_55	207	1.359	0.669	2.03	0.042*	0.047	2.671
time_quarter_56	233	0.232	0.641	0.362	0.718	-1.024	1.487
time_quarter_57	335	-0.308	0.557	-0.553	0.58	-1.4	0.784
time_quarter_58	362	-0.188	0.533	-0.352	0.725	-1.233	0.857
time_quarter_59	350	0.165	0.536	0.308	0.758	-0.886	1.216
time_quarter_60	251	-0.393	0.637	-0.617	0.537	-1.641	0.855
time_quarter_61	209	-0.351	0.674	-0.521	0.602	-1.672	0.97
time_quarter_62	233	-0.374	0.65	-0.576	0.564	-1.648	0.899
time_quarter_63	239	1.966	0.657	2.991	0.003*	0.678	3.255
time_quarter_64	241	2.181	0.649	3.358	0.001*	0.908	3.453
time_quarter_65	305	1.989	0.581	3.422	0.001*	0.85	3.127
time_quarter_66	337	0.292	0.563	0.518	0.604	-0.813	1.396
time_quarter_67	397	0.067	0.53	0.126	0.899	-0.972	1.106
time_quarter_68	299	0.459	0.591	0.777	0.437	-0.7	1.618
time_quarter_69	270	0.844	0.61	1.384	0.166	-0.351	2.038

Variable	Support	Coef.	Std.Err.	z	P>|z|	0.025	0.975
time_quarter_70	286	2.833	0.581	4.879	0*	1.695	3.972
time_quarter_71	424	2.763	0.514	5.379	0*	1.756	3.77
time_quarter_72	616	-1.051	0.458	-2.292	0.022*	-1.949	-0.152
time_quarter_73	505	-3.239	0.485	-6.682	0*	-4.19	-2.289
time_quarter_74	374	-5.897	0.551	-10.694	0*	-6.978	-4.816
time_quarter_75	204	-1.262	0.691	-1.826	0.068	-2.617	0.092
time_quarter_76	196	0.362	0.705	0.514	0.607	-1.02	1.744
time_quarter_77	170	1.178	0.759	1.552	0.121	-0.31	2.665
time_quarter_78	124	1.094	0.862	1.268	0.205	-0.596	2.784
time_quarter_79	119	-0.987	0.878	-1.123	0.261	-2.708	0.735
time_quarter_80	102	-2.141	0.943	-2.27	0.023*	-3.99	-0.292
time_quarter_81	84	-1.773	1.033	-1.717	0.086	-3.798	0.251
time_quarter_82	205	1.932	0.68	2.843	0.004*	0.6	3.264
time_quarter_83	214	2.638	0.666	3.962	0*	1.333	3.944
time_quarter_84	202	2.285	0.685	3.337	0.001*	0.943	3.628
time_quarter_85	79	-1.078	1.067	-1.011	0.312	-3.169	1.013
time_quarter_86	86	-1.577	1.028	-1.534	0.125	-3.591	0.437
time_quarter_87	70	-3.625	1.141	-3.177	0.001*	-5.861	-1.389
time_quarter_88	61	-1.173	1.224	-0.958	0.338	-3.573	1.227

Variable	Support	Coef.	Std.Err.	z	P>|z|	0.025	0.975
time_quarter_89	37	-0.229	1.553	-0.147	0.883	-3.272	2.814
time_quarter_90	57	1.673	1.27	1.317	0.188	-0.816	4.161
time_quarter_91	60	-0.116	1.233	-0.094	0.925	-2.533	2.301
time_quarter_92	220	-0.226	1.196	-0.189	0.85	-2.569	2.117
time_quarter_93	193	-2.09	1.554	-1.345	0.179	-5.136	0.956
time_quarter_94	180	2.601	1.472	1.767	0.077	-0.285	5.487
time_quarter_95	26	4.62	1.844	2.505	0.012*	1.005	8.234

*p<0.05, indicates the correlation between this variable and location clustering label is significant.

The weighted significance rates, weighted by support value, from time_quarter_0 to time_quarter_95 of this table is computed as 47.39%.

Supplementary Table 4 Mixed-Effects Model Results: Association Between Temporal Features and DBSCAN Location Clustering Labels, From the Non-smoking Events with Quarter-Hour Intervals

Variable	Support	Coef.	Std.Err.	z	P>|z|	0.025	0.975
day_of_week	8634	0.327	1.348	0.242	0.809	-2.316	2.97
is_weekend	1527	-2.315	0.478	-4.847	0*	-3.251	-1.379
season	9161	-5.532	10.814	-0.512	0.609	-26.726	15.663
time_quarter_0	380	9.094	0.865	10.511	0*	7.398	10.79
time_quarter_1	274	8.871	1.011	8.772	0*	6.889	10.852
time_quarter_2	148	8.187	1.362	6.01	0*	5.517	10.857
time_quarter_3	11	-1.108	4.969	-0.223	0.824	-10.847	8.63
time_quarter_4	9	-3.58	5.51	-0.65	0.516	-14.379	7.219
time_quarter_5	10	0.593	5.216	0.114	0.91	-9.631	10.817
time_quarter_6	8	0.796	5.857	0.136	0.892	-10.683	12.274

Variable	Support	Coef.	Std.Err.	z	P>|z|	0.025	0.975
time_quarter_7	8	0.468	5.834	0.08	0.936	-10.966	11.902
time_quarter_8	10	1.641	5.231	0.314	0.754	-8.611	11.894
time_quarter_9	21	1.3	3.623	0.359	0.72	-5.802	8.402
time_quarter_10	20	1.651	3.722	0.444	0.657	-5.645	8.947
time_quarter_11	17	2.006	4.028	0.498	0.619	-5.889	9.901
time_quarter_12	10	3.816	5.214	0.732	0.464	-6.402	14.035
time_quarter_13	8	4.602	5.808	0.792	0.428	-6.782	15.986
time_quarter_14	9	3.023	5.515	0.548	0.584	-7.787	13.833
time_quarter_15	5	2.16	7.369	0.293	0.769	-12.283	16.604
time_quarter_16	7	1.333	6.344	0.21	0.834	-11.101	13.766
time_quarter_17	5	1.103	7.453	0.148	0.882	-13.504	15.71
time_quarter_18	5	2.177	7.465	0.292	0.771	-12.454	16.809
time_quarter_19	4	1.482	8.24	0.18	0.857	-14.667	17.631
time_quarter_20	13	1.648	4.606	0.358	0.72	-7.38	10.677
time_quarter_21	25	1.218	3.324	0.366	0.714	-5.297	7.733
time_quarter_22	45	1.03	2.488	0.414	0.679	-3.847	5.906
time_quarter_23	102	1.145	1.805	0.634	0.526	-2.393	4.683
time_quarter_24	172	1.814	1.719	1.055	0.291	-1.555	5.182
time_quarter_25	186	1.521	1.769	0.86	0.39	-1.945	4.988
time_quarter_26	129	0.744	1.708	0.436	0.663	-2.603	4.091
time_quarter_27	65	0.096	2.116	0.046	0.964	-4.051	4.244
time_quarter_28	38	0.843	2.696	0.313	0.754	-4.441	6.127
time_quarter_29	31	1.071	2.994	0.358	0.721	-4.797	6.94
time_quarter_30	20	0.997	3.727	0.267	0.789	-6.307	8.3
time_quarter_31	18	1.358	3.931	0.346	0.73	-6.347	9.064
time_quarter_32	105	0.297	1.674	0.178	0.859	-2.983	3.578
time_quarter_33	108	-0.663	1.648	-0.402	0.687	-3.892	2.566
time_quarter_34	105	-0.754	1.669	-0.452	0.652	-4.024	2.517

Variable	Support	Coef.	Std.Err.	z	P>|z|	0.025	0.975
time_quarter_35	138	5.395	1.41	3.825	0*	2.63	8.159
time_quarter_36	364	1.413	0.887	1.593	0.111	-0.325	3.152
time_quarter_37	542	-13.142	0.728	-18.063	0*	-14.568	-11.716
time_quarter_38	436	-17.898	0.794	-22.543	0*	-19.455	-16.342
time_quarter_39	499	-18.115	0.834	-21.727	0*	-19.749	-16.481
time_quarter_40	630	1.717	1.537	1.117	0.264	-1.295	4.729
time_quarter_41	839	3.28	0.976	3.36	0.001*	1.367	5.192
time_quarter_42	823	3.983	0.672	5.926	0*	2.666	5.301
time_quarter_43	759	5.898	0.676	8.719	0*	4.572	7.223
time_quarter_44	885	6.513	0.592	11.004	0*	5.353	7.673
time_quarter_45	997	6.829	0.566	12.07	0*	5.72	7.938
time_quarter_46	1176	8.013	0.539	14.87	0*	6.957	9.07
time_quarter_47	1191	7.999	0.53	15.105	0*	6.961	9.037
time_quarter_48	1114	2.986	0.54	5.531	0*	1.928	4.044
time_quarter_49	977	-5.332	0.566	-9.414	0*	-6.442	-4.222
time_quarter_50	768	-18.998	0.601	-31.625	0*	-20.176	-17.821
time_quarter_51	652	-14.626	0.659	-22.189	0*	-15.918	-13.334
time_quarter_52	412	-10.327	0.832	-12.404	0*	-11.958	-8.695
time_quarter_53	613	-10.369	0.699	-14.828	0*	-11.74	-8.999
time_quarter_54	757	-9.687	0.645	-15.022	0*	-10.951	-8.423
time_quarter_55	784	-8.688	0.632	-13.752	0*	-9.926	-7.45
time_quarter_56	442	4.459	0.808	5.518	0*	2.875	6.042
time_quarter_57	138	4.476	1.415	3.163	0.002*	1.702	7.249
time_quarter_58	197	1.098	1.227	0.895	0.371	-1.306	3.503
time_quarter_59	255	0.826	1.097	0.753	0.451	-1.324	2.976
time_quarter_60	266	0.389	1.087	0.357	0.721	-1.742	2.52
time_quarter_61	173	-0.265	1.349	-0.196	0.844	-2.909	2.379
time_quarter_62	228	1.675	1.142	1.467	0.142	-0.563	3.913

Variable	Support	Coef.	Std.Err.	z	P>|z|	0.025	0.975
time_quarter_63	306	1.407	1.01	1.393	0.163	-0.572	3.386
time_quarter_64	346	1.625	0.959	1.694	0.09	-0.256	3.505
time_quarter_65	321	0.248	1.006	0.247	0.805	-1.723	2.219
time_quarter_66	285	0.423	1.044	0.405	0.685	-1.623	2.469
time_quarter_67	312	0.064	1.017	0.063	0.95	-1.93	2.057
time_quarter_68	310	-0.741	1.029	-0.72	0.472	-2.758	1.276
time_quarter_69	267	-1.077	1.169	-0.921	0.357	-3.367	1.214
time_quarter_70	166	-1.461	1.587	-0.921	0.357	-4.572	1.65
time_quarter_71	142	-1.745	1.506	-1.159	0.246	-4.697	1.206
time_quarter_72	208	-5.829	1.477	-3.947	0*	-8.723	-2.934
time_quarter_73	227	-5.141	1.394	-3.688	0*	-7.873	-2.409
time_quarter_74	172	-3.575	1.464	-2.442	0.015*	-6.444	-0.706
time_quarter_75	67	1.454	2.167	0.671	0.502	-2.793	5.702
time_quarter_76	48	2.265	2.448	0.925	0.355	-2.534	7.063
time_quarter_77	55	0.252	2.291	0.11	0.912	-4.237	4.742
time_quarter_78	76	0.271	1.957	0.139	0.89	-3.564	4.107
time_quarter_79	75	0.114	1.968	0.058	0.954	-3.743	3.972
time_quarter_80	57	0.828	2.233	0.371	0.711	-3.548	5.204
time_quarter_81	34	2.283	2.842	0.803	0.422	-3.288	7.854
time_quarter_82	125	8.407	1.652	5.089	0*	5.169	11.646
time_quarter_83	147	9.189	1.574	5.838	0*	6.104	12.274
time_quarter_84	147	7.385	1.604	4.605	0*	4.242	10.529
time_quarter_85	77	0.308	2.175	0.142	0.887	-3.955	4.572
time_quarter_86	62	-2.27	2.134	-1.064	0.288	-6.452	1.913
time_quarter_87	125	-16.456	1.575	-10.447	0*	-19.544	-13.369
time_quarter_88	200	-34.521	1.236	-27.923	0*	-36.944	-32.097
time_quarter_89	461	-10.073	0.815	-12.357	0*	-11.67	-8.475
time_quarter_90	680	0.613	0.671	0.914	0.361	-0.701	1.928

Variable	Support	Coef.	Std.Err.	z	P>|z|	0.025	0.975
time_quarter_91	876	9.274	0.597	15.543	0*	8.104	10.443
time_quarter_92	897	10.509	0.589	17.841	0*	9.355	11.664
time_quarter_93	790	10.188	0.621	16.398	0*	8.97	11.405
time_quarter_94	601	9.664	0.7	13.801	0*	8.291	11.036
time_quarter_95	427	9.223	0.819	11.264	0*	7.619	10.828

*p<0.05, indicates the correlation between this variable and location clustering labels is significant.

The weighted significance rates, weighted by support value, from time_quarter_0 to time_quarter_95 of this table is computed as 74.79%.

Supplementary Table 5 Mixed-Effects Model Results: Association Between Temporal Features and DBSCAN Location Clustering Labels of Smoking events with half-hour intervals

Variable	Support	Coef.	Std.Err.	z	P>|z|	0.025	0.975
day_of_week	4608	1.69	0.63	2.684	0.007*	0.456	2.924
is_weekend	1301	2.796	0.313	8.932	0*	2.182	3.41
season	4352	-6.424	12.939	-0.497	0.62	-31.784	18.935
time_half_hour_0	49	5.717	1.348	4.242	0*	3.075	8.358
time_half_hour_1	38	3.134	1.528	2.051	0.04*	0.139	6.128
time_half_hour_2	28	1.452	1.781	0.815	0.415	-2.039	4.944
time_half_hour_3	21	3.72	2.05	1.814	0.07	-0.298	7.738
time_half_hour_4	17	4.462	2.29	1.949	0.051	-0.026	8.951
time_half_hour_5	39	4.128	1.512	2.73	0.006*	1.164	7.092
time_half_hour_6	39	4.828	1.511	3.195	0.001*	1.866	7.79
time_half_hour_7	13	4.721	2.599	1.817	0.069	-0.372	9.814
time_half_hour_8	3	5.909	5.418	1.091	0.275	-4.71	16.528
time_half_hour_9	19	9.391	2.161	4.345	0*	5.154	13.627
time_half_hour_10	56	7.785	1.27	6.13	0*	5.296	10.274
time_half_hour_11	157	1.32	0.772	1.709	0.087	-0.194	2.833

Variable	Support	Coef.	Std.Err.	z	P>|z|	0.025	0.975
time_half_hour_12	339	-0.246	0.603	-0.408	0.683	-1.427	0.935
time_half_hour_13	340	1.173	0.575	2.04	0.041*	0.046	2.3
time_half_hour_14	184	2.56	0.711	3.6	0*	1.166	3.953
time_half_hour_15	141	1.734	0.817	2.123	0.034*	0.133	3.334
time_half_hour_16	188	1.377	0.709	1.944	0.052*	-0.011	2.766
time_half_hour_17	264	1.545	0.611	2.529	0.011*	0.348	2.743
time_half_hour_18	249	1.283	0.63	2.038	0.042*	0.049	2.518
time_half_hour_19	172	0.927	0.749	1.237	0.216	-0.542	2.395
time_half_hour_20	182	1.45	0.723	2.005	0.045*	0.033	2.867
time_half_hour_21	297	-9.344	0.561	-16.655	0*	-10.443	-8.244
time_half_hour_22	278	-10.217	0.582	-17.56	0*	-11.358	-9.077
time_half_hour_23	261	2.501	0.602	4.155	0*	1.321	3.681
time_half_hour_24	276	0.189	0.597	0.317	0.751	-0.982	1.36
time_half_hour_25	206	-4.07	0.676	-6.024	0*	-5.394	-2.745
time_half_hour_26	261	0.471	0.614	0.767	0.443	-0.732	1.673
time_half_hour_27	311	2.14	0.561	3.816	0*	1.041	3.238
time_half_hour_28	401	0.218	0.509	0.429	0.668	-0.779	1.216
time_half_hour_29	428	-0.106	0.495	-0.215	0.83	-1.077	0.864
time_half_hour_30	325	-0.218	0.557	-0.392	0.695	-1.311	0.874
time_half_hour_31	308	1.082	0.575	1.883	0.06	-0.044	2.208
time_half_hour_32	395	1.637	0.522	3.133	0.002*	0.613	2.66
time_half_hour_33	473	0.266	0.487	0.547	0.584	-0.687	1.22
time_half_hour_34	377	0.404	0.524	0.772	0.44	-0.622	1.43
time_half_hour_35	480	2.839	0.479	5.934	0*	1.902	3.777
time_half_hour_36	657	-1.43	0.44	-3.249	0.001*	-2.293	-0.567
time_half_hour_37	474	-4.46	0.496	-9.001	0*	-5.431	-3.489
time_half_hour_38	233	0.288	0.647	0.446	0.656	-0.979	1.556
time_half_hour_39	152	-0.173	0.782	-0.221	0.825	-1.706	1.36

Variable	Support	Coef.	Std.Err.	z	P>|z|	0.025	0.975
time_half_hour_40	138	-0.757	0.813	-0.932	0.351	-2.35	0.835
time_half_hour_41	234	1.933	0.636	3.038	0.002*	0.686	3.181
time_half_hour_42	228	1.303	0.644	2.022	0.043*	0.04	2.565
time_half_hour_43	94	-2.505	0.983	-2.549	0.011*	-4.432	-0.579
time_half_hour_44	73	-0.064	1.119	-0.057	0.954	-2.258	2.13
time_half_hour_45	77	0.233	1.094	0.213	0.831	-1.91	2.377
time_half_hour_46	221	-1.172	1.188	-0.986	0.324	-3.499	1.156
time_half_hour_47	198	3.02	1.226	2.463	0.014*	0.617	5.424

*p<0.05, indicates the correlation between this variable and location clustering labels is significant.

The weighted significance rates, weighted by support value, from time_half_hour_0 to time_half_hour_47 of this table is computed as 54.96%.

Supplementary Table 6 Mixed-Effects Model Results: Association Between Temporal Features and DBSCAN Location Clustering Labels of Non-smoking events with half-hour intervals

Variable	Support	Coef.	Std.Err.	z	P>|z|	0.025	0.975
day_of_week	8634	0.327	1.348	0.242	0.809	-2.316	2.97
is_weekend	1527	-2.315	0.478	-4.847	0*	-3.251	-1.379
season	9161	-5.532	10.814	-0.512	0.609	-26.726	15.663
time_half_hour_0	386	9.031	0.859	10.515	0*	7.347	10.714
time_half_hour_1	151	8.028	1.349	5.953	0*	5.384	10.671
time_half_hour_2	12	-2.667	4.764	-0.56	0.576	-12.004	6.67
time_half_hour_3	12	0.754	4.806	0.157	0.875	-8.666	10.174
time_half_hour_4	22	1.291	3.542	0.365	0.715	-5.651	8.233

Variable	Support	Coef.	Std.Err.	z	P>|z|	0.025	0.975
time_half_hour_5	22	2.138	3.544	0.603	0.546	-4.807	9.084
time_half_hour_6	11	4.097	4.968	0.825	0.41	-5.641	13.834
time_half_hour_7	10	2.719	5.227	0.52	0.603	-7.526	12.965
time_half_hour_8	8	1.188	5.917	0.201	0.841	-10.409	12.785
time_half_hour_9	7	1.61	6.287	0.256	0.798	-10.713	13.932
time_half_hour_10	26	1.384	3.268	0.424	0.672	-5.021	7.789
time_half_hour_11	112	1.201	1.716	0.7	0.484	-2.161	4.563
time_half_hour_12	208	1.448	1.631	0.888	0.375	-1.75	4.646
time_half_hour_13	148	0.895	1.632	0.548	0.583	-2.303	4.093
time_half_hour_14	41	0.88	2.601	0.338	0.735	-4.218	5.977
time_half_hour_15	27	1.168	3.225	0.362	0.717	-5.152	7.488
time_half_hour_16	116	-0.532	1.588	-0.335	0.738	-3.646	2.581
time_half_hour_17	228	3.401	1.114	3.054	0.002*	1.219	5.584
time_half_hour_18	546	-13.033	0.725	-17.98	0*	-14.453	-11.612
time_half_hour_19	736	-12.135	0.671	-18.083	0*	-13.45	-10.819
time_half_hour_20	856	3.215	0.957	3.36	0.001*	1.34	5.09
time_half_hour_21	1072	4.474	0.595	7.517	0*	3.308	5.641
time_half_hour_22	1281	7.158	0.507	14.112	0*	6.164	8.153
time_half_hour_23	1543	8.781	0.484	18.138	0*	7.832	9.73

Variable	Support	Coef.	Std.Err.	z	P>|z|	0.025	0.975
time_half_hour_24	1405	-1.643	0.493	-3.331	0.001*	-2.609	-0.676
time_half_hour_25	971	-13.276	0.554	-23.955	0*	-14.363	-12.19
time_half_hour_26	771	-17.067	0.608	-28.069	0*	-18.259	-15.875
time_half_hour_27	835	-8.115	0.616	-13.166	0*	-9.323	-6.907
time_half_hour_28	485	4.221	0.773	5.464	0*	2.707	5.735
time_half_hour_29	272	1.018	1.062	0.959	0.338	-1.063	3.099
time_half_hour_30	310	0.129	1.016	0.127	0.899	-1.863	2.121
time_half_hour_31	360	1.125	0.943	1.193	0.233	-0.724	2.973
time_half_hour_32	451	1.321	0.845	1.562	0.118	-0.336	2.977
time_half_hour_33	396	0.211	0.931	0.226	0.821	-1.613	2.035
time_half_hour_34	363	-0.653	0.991	-0.659	0.51	-2.595	1.289
time_half_hour_35	245	-2.493	1.312	-1.9	0.057	-5.065	0.079
time_half_hour_36	237	-4.718	1.352	-3.49	0*	-7.367	-2.068
time_half_hour_37	186	-3.033	1.396	-2.172	0.03*	-5.77	-0.296
time_half_hour_38	79	0.576	1.928	0.299	0.765	-3.202	4.354
time_half_hour_39	85	0.647	1.857	0.349	0.727	-2.992	4.286
time_half_hour_40	69	1.724	2.031	0.849	0.396	-2.257	5.705
time_half_hour_41	160	8.054	1.492	5.4	0*	5.13	10.977
time_half_hour_42	177	6.018	1.422	4.232	0*	3.231	8.805

Variable	Support	Coef.	Std.Err.	z	P>|z|	0.025	0.975
time_half_hour_43	137	-16.386	1.506	-10.88	0*	-19.338	-13.434
time_half_hour_44	481	-9.649	0.797	-12.1	0*	-11.211	-8.086
time_half_hour_45	981	3.722	0.575	6.471	0*	2.595	4.85
time_half_hour_46	1084	10.913	0.544	20.043	0*	9.846	11.98
time_half_hour_47	729	9.896	0.642	15.404	0*	8.637	11.155

*$p<0.05$, indicates the correlation between this variable and location clustering labels is significant.

The weighted significance rates, weighted by support value, from time_half_hour_0 to time_half_hour_47 of this table is computed as 81.90%.
